# Utilization of psychiatric rehabilitation services and influencing factors among people with psychotic disorders in rural communities of Guangxi, China

**DOI:** 10.1186/s13033-018-0197-z

**Published:** 2018-04-17

**Authors:** Hongye Luo, Edward B. McNeil, Qiming Feng, Hongheng Li, Qiang Chen, Xianjing Qin, Jun Feng, Sawitri Assanangkornchai

**Affiliations:** 1 Information and Management School, Guangxi Medical University, Nanning, Guangxi Zhuang Autonomous Region China; 2 Epidemiology Unit, Faculty of Medicine, Prince of Songkla University, Hat Yai, Songkhla Thailand; 3 Guangxi Brain Hospital, Liuzhou, Guangxi Zhuang Autonomous Region China

**Keywords:** Severe mental disorder, People with psychotic disorders, Psychiatric rehabilitation services, Influencing factors, Rural communities

## Abstract

**Objectives:**

To identify the rate and predictors of utilization of rehabilitation services among people with psychotic disorders in rural communities of Guangxi.

**Study design:**

A cross-sectional survey was conducted among individuals with schizophrenia or other psychoses (severe mental disorder, ICD10: F20–F29), aged over 15 years, and their care-givers in Guangxi, China. Trained village doctors located individuals known to them and suspected as having schizophrenia or other psychoses within the target areas and recruited them into the study. Data on demographic characteristics, clinical symptoms and functions, treatment history, and reasons, if any, for non-utilization of mental health service were collected. Logistic regression was used to determine associated factors for utilization of mental health services.

**Results:**

A total of 424 individuals experiencing psychosis (mean age 41.4 ± 13.0 years, 60.6% male) and 319 caregivers (mean age 55.3 ± 14.2 years) were interviewed. The median duration of disease was 13.4 years. 83.0% of patients had never used rehabilitation services. Greater use of rehabilitation was associated with having a non-organic disorder (OR = 11.6, 95% CI = 1.6–86.0) and living with a caregivers (OR = 3.2, 95% CI = 1.2–8.3). The top three reasons for not using rehabilitation services were lack of awareness (57.1%), lack of money (14.2%) and lack of belief in the service (12.8%).

**Conclusions:**

These findings indicate a high unmet need for psychiatric rehabilitation services among people with psychotic disorders in rural areas of Guangxi. Strategies such as outreach programme and collaborative and partnership network with the local community are needed to encourage people with psychotic disorders in rural communities to increase their utilization of rehabilitation services.

## Background

Mental disorders occur at high rates in every country, and have already become a global challenge, especially in fast-growing developing countries such as China [1, 2]. The second national schizophrenic epidemiological investigation of China in 1993 showed that more than 83 million were living with mental disorders, 16 million suffered from severe mental disorders such as schizophrenia, and two-thirds lived in rural areas [3].

Psychiatric rehabilitation is an important part of a mental health service. It promotes recovery, community integration, and improves quality of life for patients who have been diagnosed with any mental health condition that seriously impairs their ability to lead meaningful lives. If the patients received rehabilitation their social function will be improved which in turn will decrease the burden of caregivers [4]. Psychiatric rehabilitation services focus on helping patients develop skills and access resources needed to increase their ability to be successful and satisfied in the living, working, learning, and social environments of their choice [5]. They are especially related to the improvement of functioning and quality of life [6]. Additionally, psychiatric rehabilitation aims to reintegrate patients with severe mental disorders into society and help them to live fulfilling lives [7, 8]. Psychiatric rehabilitation services include medications for treatment-resistant psychosis and affective disorders, physical health promotion, psychological interventions such as cognitive behavioral therapy and family interventions, occupational therapy, and supported employment [9].

The practice of psychiatric rehabilitation over the last few decades has undergone gradual refinement and sophistication, which has resulted in a multitude of intervention programmes in the United States, Canada and the United Kingdom [9–12]. However, to a large extent, it has been restricted to developed countries. Its importance has not been recognized in many developing countries, and its practice is still rare compared to the use of medicine to treat patients. Although mental health is part of the primary healthcare system, very little active psychiatric treatment or psychiatric rehabilitation happens especial in most low- and middle-income countries [6]. Evidently, large differences between countries may exist with respect to the availability and organization of rehabilitation services although most jurisdictions would argue that not all people living with severe mental disorders such as schizophrenia or delusional disorders require rehabilitation at any one time [13]. There is little in terms of documentation of their activities and limited research on the utilization of psychiatric rehabilitation services in low- and middle-income countries [6]. According to previous studies, 40% of patients with schizophrenia did not have any vocational or educational perspectives at discharge from hospital in Belgium [14, 15]. Studies conducted in Israel showed only about 15–20% of the estimated number of the eligible population received the service [16, 17]. The report of Nepal showed 1–20% of people with psychotic disorders in outpatient facilities received psychiatric rehabilitation service [18, 19].

Lack of utilization of mental health services can lead to irreversible conditions. However, mental health services in China are mostly provided by psychiatric specialists in large hospitals. In addition, most psychiatrists and psychiatric specialist hospitals are located in cities. Guangxi is located in southern China. Very few mental health services exist in this relatively underdeveloped province, especially in rural areas. The prevalence of schizophrenia in rural Guangxi was 9.2% [20, 21]. Utilization of mental health services in rural communities of Guangxi may be inadequate.

WHO stated there remains a group of patients with psychotic disorders who do not recover adequately to be discharged home following efficient psychiatric services [18]. Lack of access to high-quality services also exists in the wealthiest countries. In the USA, less than 5% of people with severe psychiatric disorders can access high-quality psychiatric rehabilitation [10]. It has been estimated that around 1% of people in England with schizophrenia received inpatient rehabilitation at any one time [11]. It showed that rehabilitation services are far from generally available. A study in China reported that the utilization rate of psychiatric rehabilitation services by patients with schizophrenia was only 1.4%, and most of the patients never continued with the service once they went back into the community [22]. Another study in Shanghai reported that 9.2% of patients with severe mental disorders received psychiatric rehabilitation [23].

Very few surveys have been conducted attempting to understand the situation of psychiatric rehabilitation service utilization [14, 20–27], thus, little information is available on the predictors of these services, particularly community-based ones among patients with severe mental disorders. One study reported that family’s expectations and patient’s negative symptoms significantly correlated with rehabilitation needs and utilization [28]. Two other studies found that age, family structure, shame of disease and self-consciousness were associated with psychiatric rehabilitation utilization, and lack of district rehabilitation facility resources and personnel influenced the provision of psychiatric rehabilitation services [29, 30].

Over half of the people with schizophrenia are not receiving proper care [2]. Absence of a national mental health policy or programme in many LAMIC makes it unrealistic to expect that psychosocial interventions would be high on their agenda. It is therefore crucial for the government to formulate precise mental health policies to guide the practices to address these pressing issues [31]. Based on this point, the national mental health work plan of China (2015–2020) explicitly states that efforts are needed to improve psychiatric rehabilitation services. Every province must establish and develop community-based rehabilitation programs for mental disability patients. The plan demands at least one mental health service center be built in every county. As an important part of the mental health service system, county mental health center functions should cover the provision of psychiatric rehabilitation services [32]. Concerns about the levels of rehabilitation service received by those with serious mental illness have been growing as a result of recent changes in social welfare policy and mental health care delivery systems [22]. Assessing the utilization forms an important constituent in the planning of Mental Health Services [23]. Identifying the current psychiatric rehabilitation utilization rate and related barriers is a critical first step in planning, developing and targeting interventions to address the emerging needs of people with psychotic disorders and improve the appropriateness of psychiatric rehabilitation service and health outcomes of those with serious mental illness.

According to the current status of psychiatric rehabilitation research [14, 15, 33, 34], two questions are addressed: (1) what are the rates of psychiatric rehabilitation service utilization among persons with severe mental disorders? And (2) what factors are associated with the utilization of psychiatric rehabilitation services among these patients? In this study, we sought to improve our understanding of the utilization of psychiatric rehabilitation services among persons with severe mental disorders. Results of this study can be used to assist policy makers formulate more appropriate mental health strategies in Guangxi Zhuang Autonomous Region. Patient’s symptoms have previously been shown to be significantly correlated with rehabilitation needs [2, 30, 31]. Thus, the relationship between the uses of psychiatric rehabilitation services with patient symptoms was also examined.

## Methods

### Sample selection

A cross-sectional survey was conducted to investigate the utilization of psychiatric rehabilitation among individuals aged over 15 years with severe mental disorders and their caregivers during June to September, 2016. The survey employed a multistage stratified, clustered sampling scheme to select 118 villages from nine towns in three counties of Guangxi Autonomous Region, China. Altogether, 12 psychiatrists, 36 township hospital nurses or doctors, 118 village doctors and 20 research assistants were trained and participated in the survey. Liujiang county, Liucheng county and Xiangzhou county were selected as sample counties. Three townships were selected from each county based on the distance and population size. Four most populous administrative villages were chosen from each township (If the village population is less than 1000, the neighbor village was chosen to increase the population). All the natural villages of the administrative village were included in the sample. Finally 118 villages from three counties were included in this study, including 36 villages from Lijiang county, 41 villages from Liucheng county and 40 villages from Xiangzhou county with 47,929 residents altogether. All individuals suspected as having schizophrenia or other psychoses within the target area reported by the village doctors were screened by the township nurses and diagnosed by the psychiatrists. A total of 424 severe mental disorder patients were diagnosed and interviewed, including 185 patients from Liujiang county, 115 patients from Liucheng county and 124 patients from Xiangzhou county.

An indigenous field worker sampling method was employed in which village doctors were trained to recruit participants. These village doctors located individuals known to them and suspected as having schizophrenia or other psychoses within the target area and recruit them into the study. Multiple sites and recruitment networks were chosen to ensure a wide coverage of the target population and to reduce volunteer bias.

### Study participants

Individuals aged 15 years or over, residing in the study area for at least 6 months prior to the study period were eligible. In this study, severe mental disorders included schizophrenia, schizotypal, and delusional disorders (International Statistical Classification of Diseases and Related Health Problems (ICD-10) codes F20–F29) and other psychoses (e.g. organic psychoses) which are consistent with the “Severe mental illness treatment and management specification” launched by the Chinese Government. All patients were assessed for a definitive diagnosis by qualified psychiatrists.

### Interview and measures

After providing written informed consent, each participant met in person with a research staff for an interview that lasted approximately half an hour. Data on demographic characteristics, clinical symptoms, treatment history, use of psychiatric rehabilitation services and, if appropriate, reasons for not utilizing the service were elicited. Psychiatrists conducted clinical interviews to evaluate the severity of a patient’s condition using the positive and negative syndrome scale (PANSS) [35]. Scores range from 7 to 49 for positive and negative scales and from 16 to 112 for the general scale and higher scores represent more severe symptoms. The psychiatrists also inquired about the caregiver’s information such as age, gender, education level and marital status. Each participant was given $10 as compensation for their time.

Utilization of psychiatric rehabilitation services was defined as any use of psychiatric rehabilitation programmes by the patients within 12 months prior to the survey. This included social skills training, cognitive/cognitive-behavioral therapy, family psycho education and vocational rehabilitation. In this study, the utilization of psychiatric rehabilitation services included both inpatient and community-based services.

### Ethics

The study was approved by the Ethics Committee for Research in Human Subjects, Faculty of Medicine, Prince of Songkla University. All participants provided written informed consent to participate in the study. Data collection was conducted in private and participants were assured that any information disclosed would be treated in strict confidence. All data on patients were saved and analyzed anonymously. The China Medical Board approved the study.

### Data analysis

Descriptive statistics were used to summarize the demographic data of the participants and their caregivers. Pearson’s Chi square test was used to compare differences in socio-demographic variables, cause of illness and disease duration between those utilizing and not utilizing psychiatric rehabilitation services while Student’s independent t-test was used to compare differences in positive, negative and general symptom scores. Based on some previous studies, patients’ characteristics, including demographics, course of illness, type of symptoms (positive or negative) and type of psychotic disorders as well as caregivers’ characteristics were associated with the use of treatment and rehabilitation services [21], we therefore looked at the associations between each of these variables in the univariate analyses. The variables with *p* value ≤ 0.20 in the univariate analyses were included in the initial model. To identify predictive factors influencing the utilization of psychiatric rehabilitation services, multivariate logistic regression models were then used to calculate adjusted odd ratios (OR) and 95% confidence intervals (CI). The significance level was set at p < 0.05. All data were analyzed using R version 3.3.2.

## Results

A total of 424 severe mental disorder patients (60.6% male) aged over 15 years and 319 caregivers were interviewed. The mean age of the patients was 41.4 ± 13.0 years and the caregiver was 55.3 ± 14.2 years. Most (72.4%) patients were Zhuang ethnicity, completed elementary (38.4%) or junior high school (37.7%) and 49.5% were single. 63.4% were engaged in agricultural work while 30.0% were not currently employed and 44.8% had a low per-capita annual family income level (< 981 USD). The median duration of illness was 13.4 (Interquartile range (IQR): 6.45–67) years. Median scores of positive, negative and general symptoms of the PANSS were 12, 15 and 25, respectively. The utilization rate for any rehabilitation service was 17.0%. Demographic variables are summarized in Table 1.

**Table 1:** Socio-demographic and clinical characteristics of the participants and their caregivers

Characteristic	Frequency	Percent
Gender		
Male	257	60.6
Female	167	39.4
Ethnicity		
Zhuang	307	72.4
Han and other	117	27.6
Age group (years)		
< 30	91	21.5
30–39	124	29.3
40–49	113	26.7
≥ 50	96	22.6
Education level		
Illiterate	67	15.8
Elementary school	163	38.4
Junior high school	160	37.7
High school or higher	34	8.0
Marital status		
Single	210	49.5
Married	187	44.1
Divorced or widowed	27	6.4
Occupation		
Unemployed	127	30.0
Farmer	269	63.4
Other	28	6.6
Annual family income level (US dollars)		
High (> 1802)	74	17.5
Middle (981–1802)	160	37.7
Low (< 981)	190	44.8
Cause of severe mental disease		
Organic psychoses	50	11.8
Other non-organic severe mental disorders	374	88.2
Disease duration (years)		
< 10	158	37.3
10–19	131	30.9
≥ 20	135	31.8
PANSS score, median (IQR)		
Positive scale	12 (7–14)	
Negative scale	15 (7–48)	
General psychopathology	25 (16–76)	
Caregiver		
Yes	352	83.0
No	72	17.0
Caregiver’s gender		
Male	195	61.1
Female	124	38.9

*PANSS* positive and negative syndrome scale, *SD* standard deviation

There were no significant differences between those who ever utilized and those who had never utilized any rehabilitation service in terms of gender, ethnicity, age group, marital status, occupation, annual family income, disease duration and caregiver’s gender. However, statistically significant differences were found in educational level, type of psychoses, having a caregiver and PANSS scores in the univariate analyses as shown in Table 2. Patients with schizophrenia and other non-organic mental disorders utilized psychiatric rehabilitation services more than those with organic severe mental disorders (p = 0.005). Patients who had a caregiver used the service more than those without a caregiver (p = 0.037). In addition, those who did not utilize psychiatric rehabilitation services had significantly higher positive symptoms (p = 0.024), negative symptoms (p = 0.016) and general symptoms (p = 0.003) from the PANSS.

**Table 2:** Comparison of factors between patients who utilized rehabilitation services and those who did not

	Utilization of services, N (%)		P-value
	No N = 352 (83%)	Yes N = 72 (17%)	
Gender			0.820
Male	212 (82.5)	45 (17.5)	
Female	140 (83.8)	27 (16.2)	
Ethnicity			0.637
Zhuang	257 (83.7)	50 (16.3)	
Han and others	95 (81.2)	22 (18.8)	
Age group (years)			0.801
< 30	73 (80.2)	18 (19.8)	
30–39	104 (83.9)	20 (16.1)	
40–49	93 (82.3)	20 (17.7)	
≥ 50	82 (85.4)	14 (14.6)	
Education level			0.048
Illiterate	63 (94.03)	4 (5.97)	
Elementary school	129 (79.1)	34 (20.2)	
Junior high school	133 (83.1)	27 (16.9)	
High school or higher	27 (79.4)	7 (20.6)	
Marital status			0.562
Unmarried	171 (81.4)	39 (18.6)	
Married	157 (84.0)	30 (16.0)	
Divorced or widowed	24 (88.9)	3 (11.1)	
Occupation			0.358
Unemployed	110 (86.6)	17 (13.4)	
Farmer	218 (81.0)	51 (19.0)	
Others	24 (85.7)	4 (14.3)	
Annual family income level (US dollars)			0.536
High (> 1802)	155 (81.6)	35 (18.4)	
Middle (981–1802)	60 (81.1)	14 (18.9)	
Low (< 981)	137 (85.6)	23 (14.4)	
Cause of disease			0.005
Organic psychoses	49 (98.0)	1 (2.0)	
Other non-organic severe mental disorders^†^	303 (81.0)	71 (19.0)	
Disease duration (years)			0.083
< 10	128 (81.0)	30 (19.0)	
10–19	104 (79.4)	27 (20.6)	
≥ 20	120 (88.9)	15 (11.1)	
Caregiver			0.037
Yes	290 (82.4)	62 (17.6)	
No	67 (93.1)	5 (6.9)	
Caregiver’s gender			0.264
Male	171 (87.7)	24 (12.3)	
Female	105 (84.7)	19 (15.3)	
PANSS score, median (IQR)			
Positive scale	13 (7–22)	9 (7–18)	0.024
Negative scale	17 (10–25)	13 (10–19)	0.016
General psychopathology	27 (20–37)	23 (19–28)	0.003

*PANSS* positive and negative syndrome scale, *SD* standard deviation, *IQR* interquartile range

^†^Schizophrenia and other non-organic mental disorders

From the results of the multivariate logistic regression model shown in Table 3, two factors were associated with utilization of rehabilitation: having a non-organic mental disorder (OR = 11.6, 95% CI = 1.6, 86.0) and having a caregiver (OR = 3.2, 95% CI = 1.2, 8.3).

**Table 3:** Logistic regression model presenting predictors of psychiatric rehabilitation service utilization among severe mental disorder patients

	Crude OR (95% CI)	Adjusted OR (95% CI)
Female gender (vs male)	0.9 (0.5–1.5)	
Zhuang ethnicity (vs Han and others)	0.8 (0.5–1.5)	
Age group (years): ref. = < 30		
30–39	0.8 (0.4–1.6)	
40–49	0.9 (0.4–1.8)	
≥ 50	0.7 (0.3–1.5)	
Education level: ref. = Illiterate		
Elementary school	4.2 (1.4–12.2)	
Junior high school	3.2 (1.1–9.5)	
High school or higher	4.1 (1.1–15.1)	
Marital status: ref. = unmarried		
Married	0.8 (0.5–1.4)	
Divorced or widowed	0.6 (0.2–1.9)	
Occupation: ref. = unemployed		
Farmer	1.5 (0.8–2.7)	
Other	1.1 (0.3–3.5)	
Annual family income level: ref. = low		
Middle	0.7 (0.4–1.3)	
High	1.0 (0.5–2.1)	
Cause of disease		
Other non-organic severe mental disorders (vs organic psychoses)	11.5 (1.6–84.6)	11.6 (1.6–86.0)
Disease duration (years)	1.0 (0.9–1.0)	
PANSS score		
Positive scale	1.0 (0.9–1.0)	
Negative scale	1.0 (0.9–1.0)	
General psychopathology	1.0 (0.9–1.0)	
Caregiver		
Yes (vs no)	6.2 (2.0–18.3)	3.21 (1.2–8.3)
Caregiver’s gender		
Female (vs male)	1.3 (0.6–2.4)	

*PANSS* positive and negative syndrome scale, *OR* odds ratio, *CI* confidence interval, *ref* reference group for comparison

^†^Schizophrenia and other non-organic mental disorders

As shown in Table 4, the top three reasons for not utilizing rehabilitation services were lack of awareness (57.1%), excessive cost (14.2%) and lack of belief in the service (12.8%).

**Table 4:** Reasons for non-utilization of psychiatric rehabilitation services among severe mental disorder patients, N = 352

Reason	Frequency	Percent
Lack of awareness of the service	201	57.1
Service is too expensive	50	14.2
Lack of belief in the service	45	12.8
Refused to seek or utilize the service	36	10.2
Don’t know the location of the service	20	5.7

## Discussion

This study describes the utilization of psychiatric rehabilitation services and associated factors among people with severe mental disorder in Guangxi rural communities. The utilization rate was very low, only 17.0%, which is consistent with previous studies in China and in other developing countries (1–40%) [6, 19, 22, 23, 25–27, 36]. Psychiatric rehabilitation utilization rates are also low in developed countries [37]. Reasons for the low utilization of psychiatric rehabilitation services are multifactorial. Some of the factors in developing countries are largely similar to developed countries, such as the economic problems, defective national insurance system, high social stigma of mental illness, lack staff trained in basic principles of psychosocial rehabilitation, the aim of rehabilitation is not fully understood, lack of national mental health policy [38, 39]. Mental health care in China is still predominantly institution based and focuses mainly on medical treatment. Psychiatric rehabilitation services have not received enough attention from the government. There is a scarcity of policies that have led to effective strategies and intervention programs to support individuals seeking treatment. Guangxi is an undeveloped province of China, with a lack of mental health resources, especially personnel and training, which has led to a lack of service provision. In addition, the psychiatric rehabilitation service is not covered by the current medical insurance system, making treatment unaffordable for many patients.

Compared with previous studies in China [22, 23, 25–27], the rate of patients who used psychiatric rehabilitation services was higher in this study. Li reported a rate as low as 1.0% from a national survey [26] while Zhang Jianling reported a rate of 9.2% in a district of Shanghai [22]. One possible explanation for our higher utilization rate may be because psychiatric rehabilitation services in this study included services received in hospital, while the other studies included only community psychiatric rehabilitation services. Almost all of the patients received psychiatric rehabilitation services during their hospitalization period. This means when patients return to the community, they rarely used the psychiatric rehabilitation service available to them. Most patients with severe mental disorders now live in the community and should receive treatment from community-based services. Strengthening community based rehabilitation is thus urgently needed [30].

This study found that patients with organic psychoses were less likely to use psychiatric rehabilitation services. It is likely that this group of patients is lack of the ability to express their needs adequately or their caregivers did not believe the psychiatric rehabilitation service could work on the patients, thus did not seek psychiatric rehabilitation services [40]. Several studies found that patients and their family members were unable to express their psychiatric rehabilitation needs [6, 32, 41]. In addition, the positive capacity of people with severe mental illnesses are typically ignored by society including family members, especially for those with organic psychoses, and their families do not believe the patients can return to normal lives. Family members usually take a skeptical attitude for the treatment and rehabilitation, which often leads to the patients giving up rehabilitation service [26, 41]. Family’s ignorance towards the illness may contribute to the low utilization rate of psychiatric rehabilitation services of these patients.

Patients with caregivers were more likely to utilize rehabilitation services compared to those without. Caregivers constitute the major support system bearing the responsibility for patient care [39] and influence patients to receive services that they need. Our finding raises the important role of caregivers, who could help patients towards their needs. The local government may attempt to assign caregivers, such as a village doctor, neighbor or volunteer to help patients who live alone. It also indicates that interventions such as caregivers training should be implemented to promote caregiver supervision of the patients so that they comply with medication and receive the required rehabilitation services.

This study found that participants who did not utilize psychiatric rehabilitation services had higher scores for negative symptoms, higher scores for positive symptoms and higher scores for general psychopathology compared to those who did not utilize these services. Psychiatric rehabilitation service users often have prominent ‘negative’ symptoms that may impair their motivation and organizational skills to manage daily activities. This places them at risk of self-neglect. Many also have on-going ‘positive’ symptoms which have not responded fully to medication and can make communication and engagement difficult. They are lack of the ability to express their needs thus their caregiver and family members are required to help them to access the psychiatric rehabilitation service. However, for some reasons such as the low education level or lack of the awareness, patients and their family can’t express their needs adequately. However, those differences were not shown in the multivariate analysis, indicating that the severity of the illness was not associated with utilization after adjusting for cause of disease and presence of a caregiver. This finding was consistent with the study in China reported by Li [26]. Several studies have shown that meeting the needs of psychiatric patients might be an effective strategy to control their symptoms [28, 42]. But other studies have shown that the severity of symptoms correlates well with patients who express a need for rehabilitation [43]. Thus, whether patients with high scores for negative, positive and general symptoms express a need to have psychiatric rehabilitation requires further exploration in future studies.

The top three reasons for not utilizing psychiatric rehabilitation services were lack of awareness (57%), lack of money (14%) and lack of belief in the service (13%). It seems that the patients and their family members may not be aware of the importance of the psychiatric rehabilitation services available to them, and this represents a significant barrier for patients and their families. This lack of awareness and belief in the importance of psychiatric rehabilitation services more likely led to the low utilization rate found in this study. More efforts are needed to make mentally ill patients more aware of the importance of psychiatric rehabilitation services for their long-term recovery. Regarding the cost of treatment, psychiatric rehabilitation services are not covered by the current medical insurance system [44], which may partly explain why many patients with severe mental disorders who are covered by medical insurance still could not afford to receive treatment. Efforts are warranted to strengthen legislation that improves insurance coverage of psychiatric rehabilitation services.

Results of this study should be interpreted with the following three main limitations in mind. First, since the sample was selected from only three counties, the results may be not representative of the general situation in Guangxi. Second, an indigenous field worker sampling method was employed, thus we may have missed some less severe mental disorder patients. Since the study was a community-based survey, homeless or some institutionalized patients may have been inadvertently missed. Hence, caution should be made when generalizing these findings in formulating suggestions for mental health services or policies in Guangxi. Finally, we did not evaluate the perspective of the service providers, such as their resources and skills. Further studies exploring the effect of service providers on psychiatric rehabilitation utilization are recommended.

## Conclusion

The findings of this study highlight a low utilization of psychiatric rehabilitation services in Guangxi. It is important that the government formulate relevant mental health policies and incorporate them into public health and social policy that suits the needs of severe mental disorder patients and their families. Psychiatric rehabilitation service operates as a whole system which includes many other institutions and organizations. Therefore, the collaborative and partnership network should be developed and the links with local community resource to facilitate services should be strengthened. Psychiatric rehabilitation service worker training programs and psychiatric rehabilitation intervention programs as done in other developed countries such as US, Canada, UK and Australia [40] should be developed.
